# Factors Influencing Hypertension Prevention Behaviors in Rural Areas: A Cross-Sectional Study in Indonesia

**DOI:** 10.4314/ejhs.v35i3.2

**Published:** 2025-05

**Authors:** Zaiful Rahman, Tantut Susanto, Rondhianto

**Affiliations:** 1 Master of Nursing Program, Faculty of Nursing, Universitas Jember, Jember, Indonesia; 2 Department of Community, Family and Geriatric Nursing, Faculty of Nursing, Universitas Jember, Jember, Indonesia; 3 Department of Medical Surgical of Nursing, Faculty of Nursing, Universitas Jember, Jember, Indonesia

**Keywords:** Prevention, Hypertension, Rural Area, Preventive Behavior, Community

## Abstract

**Background:**

The prevalence of hypertension in rural communities remains high despite various preventive measures. Contributing factors to suboptimal hypertension prevention include a lack of positive attitudes, weak social norms, and the adoption of unhealthy behaviors. This study aimed to analyze the factors influencing hypertension prevention behaviors in rural communities of Indonesia.

**Methods:**

A cross-sectional study was conducted among 380 hypertension patients selected through proportional random sampling from three primary health centers with the highest rates of unmet hypertension management in Bondowoso Regency, Indonesia. A self-administered questionnaire was used to collect sociodemographic data. Additional questionnaires were utilized to assess attitudes, subjective norms, perceived behavioral control, intentions, and hypertension prevention behaviors, as developed from the Theory of Planned Behavior (TPB). Blood pressure was measured using a calibrated Omron HBP-1100 sphygmomanometer. Data analysis was performed using Structural Equation Modeling (SEM) with Partial Least Squares (PLS).

**Results:**

The blood pressure classification showed that 60.3% of respondents had stage I systolic hypertension, 72.9% had stage I diastolic hypertension, and 59.2% had abnormal Mean Arterial Pressure (MAP). Internal factors (experience) significantly influenced attitudes, subjective norms, and perceived behavioral control, while external factors (media exposure) only significantly influenced subjective norms. Both attitudes, through intention, and perceived behavioral control, both directly and through intention, significantly influenced hypertension prevention behaviors (p-value < 0.05).

**Conclusions:**

TPB can effectively explain the factors influencing hypertension prevention behaviors in rural areas. Interventions that can strengthen perceived behavioral control through education and social support are essential for improving the effectiveness of hypertension prevention programs in rural communities.

## Introduction

Hypertension, a non-communicable disease, has a high prevalence that continues to rise, influenced by several factors, including age, gender, genetics, obesity, exercise, and sleep quality (1). It is considered a serious condition because of its broad impacts, which can even result in death. Often referred to as a “silent killer,” hypertension can lead to sudden death in affected individuals (2). In rural areas, the majority of the population works in various sectors, making them more susceptible to hypertension due to work-related and environmental factors. Unhealthy lifestyle behaviors, such as a high-salt and high-fat diet, are commonly continued even in individuals already diagnosed with hypertension (3). Additionally, there is a widespread lack of awareness regarding the importance of blood pressure screening (4).

In rural areas, people generally rely on local resources and have limited access to information, technology, and health services (5). Infrastructure such as roads, communication, and electricity may be insufficient, which delays emergency responses. This difficulty in accessing health services requires healthcare workers to be creative in providing care. In more remote areas, the situation is exacerbated due to greater isolation, which may lack health facilities altogether, requiring healthcare workers to travel long distances or utilize telemedicine for consultations. Challenging geographic conditions also impede the medical evacuation process during emergencies (6). Overall, rural areas are characterized by low populations, limited infrastructure, and restricted access to health services and public facilities, with a strong reliance on agriculture and local resources (7). Thus, this study focuses on understanding the hypertension issues in rural areas by considering their unique characteristics.

According to the World Health Organization (WHO), the global prevalence of hypertension has risen to 1.3 billion adults aged 30-79 years since 1990, with two-thirds of them living in low- to middle-income countries (2). The 2018 Basic Health Research (Riskesdas) revealed that the prevalence of hypertension in Indonesia had reached 34.1%, affecting over 70 million individuals. In East Java Province, the prevalence of hypertension among individuals aged over 18 years is 21.5%. Bondowoso Regency reports the lowest achievement in hypertension patient services, with only 20% of patients receiving care compared to the provincial average of 61.1%. The prevalence of hypertension in individuals aged 18-44 has increased significantly since 2013, with a notable rise in the 18-24 age group from 8.7% to 13.2%, and a similar trend in the 25-34 and 35-44 age groups in 2018 (2).

Data from the Bondowoso Regency Health Office in 2023 indicated that the prevalence of hypertension in the area was 25.25% (202,709 individuals). Of the 25 health centers in Bondowoso, three are located in urban areas and 22 in rural areas. The most significant gaps in hypertension management were observed in three rural health centers: Sumber Wringin (44%), Tamanan (37%), and Maesan (26%). These figures highlight the suboptimal management of hypertension. The rising incidence of hypertension can be attributed to a lack of preventive behaviors in the community. Insufficient awareness of the importance of a low-salt diet is prevalent, leading to high consumption of salty and fatty foods (8). Moreover, inadequate emphasis on regular physical activity contributes to sedentary lifestyles. Limited access to and participation in blood pressure screening programs further exacerbate the problem, as individuals often do not engage in regular screenings for early detection of hypertension (9).

Hypertension prevention is crucial in reducing the global and national burden of the disease. Changing unhealthy behaviors to healthier ones is a key preventive measure (10). The Theory of Planned Behavior (TPB), proposed by Ajzen in 2005, is a useful framework for understanding behaviors related to hypertension prevention. This theory emphasizes attitudes toward behavior, subjective norms, perceived behavioral control, and intention (11). Preventive behaviors can be categorized into five levels according to Leavel and Clark: health promotion, special protection, early diagnosis, rapid treatment, disability limitation, and rehabilitation. The role of nurses as educators is critical in promoting preventive behaviors. Nurses can provide essential information on adopting healthy behaviors, fostering positive attitudes, and reinforcing social norms that support hypertension prevention. Given these considerations, this study aims to explore the factors that influence hypertension prevention behaviors in rural areas of Bondowoso Regency, Indonesia, based on the Theory of Planned Behavior.

## Materials and Methods

A descriptive analytic cross-sectional study was conducted from March to August 2024 at Sumber Wringin Health Center, Tamanan Health Center, and Maesan Health Center in Bondowoso Regency, Indonesia. The study focused on areas with the highest gaps in hypertension program implementation. These areas included Sumber Wringin Health Center (9,064 people), Tamanan Health Center (9,800 people), and Maesan Health Center (12,607 people), with a total of 31,471 hypertension patients. The sample size was calculated using a sample size formula with the following parameters: population size (N = 31,471), significance level (Z1-α / 2 = 1.96), and absolute error (0.1). The sample size determined for this study was 380 respondents.

The inclusion criteria were: (1) Hypertension patients aged over 18 years but under 45 years residing in the health center areas; and (2) Hypertension patients classified as having grade 1 or grade 2 hypertension. Exclusion criteria included: (1) Respondents who refused participation; (2) Illiterate individuals; and (3) Hypertension patients undergoing treatment, post-hospitalization, or with a history of stroke. A cluster random sampling technique was used, with data collection conducted at Integrated Development Posts for Non-Communicable Diseases (IDP-NCDs). Respondents were selected randomly from these posts and provided informed consent before participation.

Data collection involved questionnaires on sociodemographic characteristics (age, gender, education, occupation, knowledge, media exposure, and experience) and the TPB-based questionnaire, which assessed attitudes, subjective norms, perceived behavioral control, intentions, and preventive behaviors. Blood pressure was measured using a calibrated OMRON HBP-1100 digital sphygmomanometer. The recruitment process involved coordination with health center heads and nurses, who acted as enumerators. Data was collected through both face-to-face and remote means, with the researcher and enumerators providing necessary assistance to respondents.

This study obtained ethical approval from the Health Research Ethics Commission (KEPK) at the Faculty of Nursing, University of Jember (letter number 292/UN25.1.14/KEPK/2024).

## Results

As shown in Table 1, the demographic characteristics of the respondents reveal that the majority belong to the late adult age group (70.5%). Regarding gender, most respondents are female (72.6%). In terms of education level, the majority of respondents have completed senior high school (43.1%), and concerning occupation, most are unemployed or housewives (58.1%). Additionally, the majority of respondents have an adequate level of knowledge (58.2%), good experience in preventing hypertension (55.5%), and substantial exposure to media related to hypertension prevention (69.2%).

**Table 1:** Characteristics of Respondent (f=380)

Characteristics	n (%)	Mean ± SD
**Age**		
Adolescence (18 – 25 years)	26 (6.9)	
Adulthood (26 – 35 years)	86 (22.6)	
Late Adulthood (36 – 45 years)	268 (70.5)	
**Type Sex**		
Man	104 (27.4)	
Woman	276 (72.6)	
**Level Education**		
No school	20 (5.3)	
Elementary School	63 (16.6)	
Junior High School	127 (33.4)	
Senior High School	164 (43.1)	
University degree (Diploma, Bachelor, Master)	6 (1.6)	
**Work**		
No working / housewife	221 (58.1)	
Farmer	77 (20.3)	
Trader	26 (6.8)	
Self-employed	52 (13.7)	
civil servant	4 (1.1)	
**Pressure (Systolic)**		160.07±13.29
Normal (<120 mmHg)	0 (0)	
Prehypertension (120-139 mmHg)	0 (0)	
Hypertension Grade 1 (140-159 mmHg)	229 (60.3)	
Hypertension Grade 2 (≥ 160 mmHg)	151 (39.7)	
**Pressure (Diastolic)**		98.11±6.07
Normal (<80 mmHg)	0 (0)	
Prehypertension (80-89 mmHg)	0 (0)	
Hypertension Grade 1 (90-99 mmHg)	277 (72.9)	
Hypertension Grade 2 (≥ 100 mmHg)	103 (27.1)	
**Mean Arterial Pressure (MAP)**		115.13 ± 8.24
Abnormal (<70 mmHg; >100 mmHg)	225 (59.2)	
Normal (70-100 mmHg)	155 (40.8)	
**Knowledge**		15.20 ± 1.12
Good	132 (34.7)	
Enough	221 (58.2)	
Not enough	27 (7.1)	
**Experience**		25.90±3.04
Good	211 (55.5)	
Not enough	169 (44.5)	
**Media Exposure**		16.87±2.16
Good	263 (69.2)	
Not enough	117 (30.8)	

The systolic blood pressure results, categorized according to hypertension classification, indicate that the majority of respondents fall into hypertension grade 1, with 229 respondents (60.3%). For diastolic blood pressure, categorized according to hypertension classification, the average result shows that 277 respondents (72.9%) have hypertension grade 1. The Mean Arterial Pressure (MAP), categorized as per the classification, reveals that 225 respondents (59.2%) have abnormal blood pressure.

The outer model test is used to assess the validity and reliability of indicators associated with latent variables. The primary purpose of this test is to evaluate the relationship between latent variables and the indicators that measure them. The outer loading test plays a critical role in explaining how much variation in the indicators reflects the construct being measured. This test ensures that the indicators accurately represent the concept under study and are consistent in their measurement. Initial evaluation of the measurement model is based on reflective constructs, specifically convergent validity and discriminant validity. The convergent validity process begins by assessing the reliability of each item (validity indicator), indicated by the loading factor value (outer loading). An indicator is considered to meet convergent validity if it has a factor loading of greater than 0.7 (12). Subsequently, composite reliability is evaluated to test the reliability of the indicators in explaining a construct. A construct or variable is deemed reliable if the composite reliability value exceeds 0.7. Additionally, a VIF value below 5 indicates the absence of multicollinearity problems (13, 14).

In this study, the convergent validity values can be found that the SEM PLS model is described the result providing a clearer picture of the relationships among the significant variables in the study, offering deeper insights and more accurate data interpretation. Based on Appendices 5 and 6, several indicators were found to be invalid, including those related to internal factor variables (X1), such as age (X1.1), gender (X1.2), education (X1.3), and knowledge (X1.4), as well as external factor variables (X2), including occupation (X2.2) and belief in others' views as normative belief (Y2.1) for the subjective norm variable (Y2), and rehabilitation (Z1.5) for hypertension prevention behavior (Z1). According to the research criteria, there is no multicollinearity as all VIF values were below 5. The composite reliability values for most attitude in behavior (Y1), subjective norms (Y2), perceived behavioral control (Y3), intentions (Y4), and hypertension prevention behavior (Z1) variables exceeded 0.7, meeting the reliability test requirements. However, internal factors (X1) and external factors (X2) did not meet this requirement, as their values were below 0.7. The next step in evaluating convergent validity is the Average Variance Extracted (AVE) value, with values above 0.5 being strongly recommended. Based on the table, most variables did not meet the AVE threshold.

The results of convergent validity and composite reliability indicate that several variables were invalid and unreliable. Therefore, the final model was rearranged based on the test results of the initial model, which examined the factors influencing hypertension prevention behavior in rural communities in Bondowoso Regency. The results of the second measurement are presented in Table 2. Based on this table, the composite reliability values exceeded 0.7, showing that all variables passed the reliability test. The next step was to check convergent validity through the AVE values. The recommended AVE threshold is above 0.5. According to the table, all AVE values were above this threshold. These results indicate that the final model, adjusted for composite reliability and AVE indicators, is accurately represented.

**Table 2:** Evaluation of the Measurement Model in the step 2 analysis of Factors that Influencing Behavior prevention Problem Hypertension (n=380)

Variables	Sub Variables	*Loading Factor*	VIF	AVE	*Composite Reliability*	*Cronbach's alpha*
Internal factors (X1)	Experience (X1.5)	1,000	1,000	1,000	1,000	1,000
External factors (X2)	exposure (X2.1)	1,000	1,000	1,000	1,000	1,000
Attitude in behavior (Y1)	Belief behavior (Y1.1)	0.932	2,105	0.862	0.926	0.840
	Evaluation impact (Y1.2)	0.925	2,105			
Subjective norm (Y2)	Motivation behave (Y2.2)	1,000	1,000	1,000	1,000	1,000
Perception control behavior (Y3)	Belief control individual (Y3.1)	0.841	1,354	0.754	0.860	0.677
	Strength belief control behavior (Y3.2)	0.895	1,354			
Intention (Y4)	Intention (Y4.1)	1,000	1,000	1,000	1,000	1,000
Behavior prevent problem hypertension (Z1)	Promotion health (Z1.1)	0.877	2,464	0.765	0.929	0.898
	protection (Z1.2)	0.854	2,308			
	Early diagnosis, treatment fast and precise (Z1.3)	0.896	2,805			
	Restrictions disability (Z1.4)	0.871	2,548			

Note: Average Variance Extracted (AVE). VIF (Variance Inflation Factor).

The results indicated that outlines the factors influencing hypertension prevention behavior, with internal factors (X1) including experience of hypertension (X1.5) and external factors (X2) including media exposure (X2.1). The attitude in behavior (Y1) is measured by two indicators: belief in behavior (Y1.1) and evaluation of impact (Y1.2). The subjective norms (Y2) variable is assessed via motivation behavioral indicators (Y2.2). The perception of control behavior (Y3) includes belief in control (Y3.1) and strength of control beliefs (Y3.2), both influencing hypertension prevention behavior. The intention (Y4) is represented by the intention indicator (Y4.1). Meanwhile, hypertension prevention behavior (Z1) includes four indicators: health promotion (Z1.1), protection (Z1.2), early diagnosis, and treatment (Z1.3), and restriction of disability (Z1.4). All these variables are integrated into the model as they meet the established indicators, ensuring that the model accurately describes the relationships between factors influencing hypertension prevention behavior.

Discriminant validity is tested to ensure that the set of indicators is not unidimensional. This test measures the extent to which the construct is distinct from other constructs. In other words, the value of a construct must be greater than the value of other constructs. This can be assessed through the Fornell-Larcker criterion and cross-loading results (14). The Fornell-Larcker criterion asserts that the value of a latent variable must be greater than the value of any other latent variables. The finding showed that each latent variable has a higher value than the others, confirming the discriminant validity of each latent variable in the model. The cross-loading results indicated the strength of the relationship between each variable and its own indicators compared to those from other construct blocks. Strong discriminant validity is confirmed when the correlation between a construct and its indicators exceeds the correlation with indicators from other constructs. The results indicated that most indicators in each block correlate more strongly with their own variables than with those from other blocks.

After confirming convergent and discriminant validity, the next step was to evaluate the structural model (inner model). Composite reliability was used to test indicator reliability. If the composite reliability value is > 0.7, the indicator is considered reliable. As shown in Table 3, the composite reliability value in this study is > 0.7, indicating that all indicators are reliable and meet the necessary requirements to proceed to the next phase. A variable is considered valid based on the Cronbach's Alpha criterion if it has a value greater than 0.6. The results presented in Table 2 indicate that all variables in this study have a Cronbach's Alpha value above 0.6, meeting the reliability requirements.

**Table 3:** Model Fit Factors That Influence Behavior Prevention Problem Hypertension (n=380)

Model Indicator	Results	Parameter	Information
SRMR	0.062	< 0.1	Fit
d_ULS	0.304	< 0.5	Fit
d_G	0.238	< 0.382	Not Fit
*Chi Square*	585,624	>125,450	Fit
NFI	0.834	>0.9	Not Fit
RMS *Theta*	0.278	<1.02	Fit

Note: Normed Fit Index (NFI). Root Mean Square (RMS). d_ULS (dissimilarity in Unweighted Least Squares). Standardized Root Mean Square Residual (SRMR)

Structural model testing aims to test the proposed hypothesis by evaluating the internal model. This evaluation includes aspects such as model fit, coefficient of determination (R^2^), effect size (f^2^), predictive relevance (Q^2^), path coefficients, and significance testing for both direct and indirect effects of latent variables (14). The effect size (f^2^) measures the influence of an independent variable on the dependent variable. An f^2^ value of 0.02 is considered small, 0.15 is moderate, and 0.35 is large. The effect size results from this study indicated significant contributions from several variables, including:

- Internal factors on attitudes in behavior (f^2^ = 1.004 > 0.35), indicating a large effect.
- Internal factors on subjective norms (f^2^ = 0.466 > 0.35), indicating a large effect.
- Internal factors on perceived behavioral control (f^2^ = 0.861 > 0.35), indicating a large effect.
- External factors on subjective norms (f^2^ = 0.029 < 0.15), indicating a small effect.
- Attitudes in behavior towards intention (f^2^ = 0.314 < 0.35), indicating a moderate effect.
- Attitudes in behavior towards behavior to prevent hypertension (f^2^ = 0.293 < 0.35), indicating a moderate effect.
- Subjective norms towards intention (f^2^ = 0.022 < 0.15), indicating a small effect.
- Perceived behavioral control towards intention (f^2^ = 0.050 < 0.15), indicating a small effect.
- Perceived behavioral control towards behavior to prevent hypertension (f^2^ = 0.162 < 0.35), indicating a moderate effect.
- Intention towards behavior to prevent hypertension (f^2^ = 0.163 < 0.35), indicating a moderate effect.
- The R Square (R^2^) values indicate the strength of the model, with values greater than 0.75 indicating strong strength, values above 0.50 indicating moderate strength, and values above 0.25 indicating weak strength. The adjusted R^2^ provides a more accurate measure of a model's explanatory power. The R^2^ values in this study described that:
  - Internal and external factors on attitudes in behavior (R^2^ = 0.501), indicating moderate strength (50.1%).
  - Internal and external factors on subjective norms (R^2^ = 0.318), indicating weak strength (31.8%).
  - Internal and external factors on perceived behavioral control (R^2^ = 0.463), indicating weak strength (46.3%).
  - Internal and external factors, attitudes in behavior, subjective norms, and perceived behavioral control on intentions (R^2^ = 0.629), indicating moderate strength (62.9%).
  - Internal and external factors, attitudes in behavior, subjective norms, perceived behavioral control, and intentions on behavior to prevent hypertension (R^2^ = 0.783), indicating strong strength (78.3%).

The Q^2^ values indicate the predictive relevance of the model, with values of 0.02 (weak), 0.15 (moderate), and 0.35 (large) (15, 16). The Q^2^ values in this study demonstrated that internal and external factors together can explain variations in attitudes in behavior with high predictive ability (Q^2^ = 0.427), subjective norms with moderate predictive ability (Q^2^ = 0.311), perceived behavioral control with moderate predictive ability (Q^2^ = 0.347), intentions with great predictive ability (Q^2^ = 0.613), and behavior to prevent hypertension problems with great predictive ability (Q^2^ = 0.591). Since the Q^2^ values are all above zero, the model demonstrates good predictive relevance.

**Figure 1:**
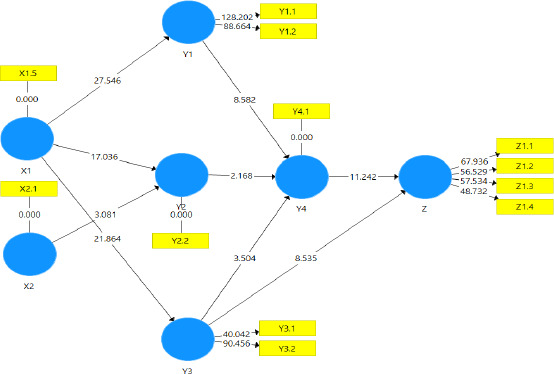
Final Fit Model of Factors Influencing Prevention Behavior of Hypertension Note:
X1: Internal FactorsX1.5: ExperienceY1: Attitude in behaviorY1.1: Belief behaviorY1.2: Evaluation impactY2: Subjective normY2.2: Motivation behaviorY3: Perception control behaviorY3.1: Confidence control individualY3.2: Strength of behavioral control beliefsY4.1: IntentionX2: External FactorsX2.1: Media exposureZ1: Behavior prevent problem hypertensionZ1.1: Health promotionZ1.2: Specific protectionZ1.3: Early diagnosis, treatment fast and preciseZ1.4: Rehabilitation

Figure 1 described that model fit was assessed using the Standardized Root Mean Square Residual (SRMR), which measures the average difference between observed and predicted correlations. An SRMR value of less than 0.1 indicates a good fit. Other indicators, including d_ULS, d_G, and the Chi-Square, were also used. The results, shown in Table 3, indicate that all values met the criteria for a good fit.

Hypothesis testing analyzed both direct and indirect effects. Table 4 showed that internal factors significantly influence attitudes in behavior, subjective norms, and perceptions of behavioral control. External factors significantly influence subjective norms. Attitudes in behavior and perceived behavioral control, mediated by intention, significantly influenced hypertension prevention behavior. However, external factors did not directly affect attitudes in behavior or perceptions of behavioral control.

**Table 4:** Results of Hypothesis Testing of Influencing Factors Behavior Prevention Problem Hypertension (n=380)

Hypothesis	*Original Sample* (O)	*Sample Mean* (M)	*Standard Deviation* (STDEV)	*T Statistics* (|O/STDEV|)	*P Values*
***Direct Effect***					
Internal factors towards attitude in behavior	0.722	0.722	0.028	25,436	0.0001
factors towards subjective norms	0.587	0.585	0.034	17,112	0.0001
Internal factors towards perception control behavior	0.658	0.658	0.031	21,489	0.0001
External factors to attitude in behavior	-0.051	-0.055	0.037	1,385	**0.167**
factors on subjective norms	-0.145	-0.143	0.045	3,191	0.002
External factors to perception control behavior	0.078	0.077	0.043	1,804	**0.072**
Attitude in behavior towards intention	0.540	0.549	0.064	8,472	0.0001
Subjective norms towards intention	0.140	0.135	0.067	2,091	0.037
Perception control behavior towards intention	0.191	0.185	0.056	3,420	0.001
Perception control behavior towards behavior prevent problem hypertension	0.260	0.261	0.038	6,867	0.0001
Intention towards behavior prevent problem hypertension	0.306	0.303	0.047	6,489	0.0001
***Indirect Effect***					
Internal factors towards intention	0.598	0.598	0.030	19,852	0.0001
Internal factors towards behavior prevent problem hypertension	0.658	0.658	0.025	26,084	0.0001
External factors on intention	-0.033	-0.036	0.029	1,131	**0.258**
External factors to behavior prevent problem hypertension	-0.011	-0.013	0.030	0.373	**0.710**
Attitude in behavior through intention towards behavior prevent problem hypertension	0.165	0.166	0.030	5,578	0.0001
Subjective norms through intention towards behavior prevent problem hypertension	0.043	0.042	0.023	1,879	**0.061**
Perception control behavior through intention towards behavior prevent problem hypertension	0.058	0.056	0.018	3,170	0.002

Note. Internal factors are all variables that are inherent in the subject, such as age, gender, education level, knowledge of hypertension, and experiences of hypertension. While external factors are all kinds of variables that exist outside the subject, such as exposure of media and job or occupation.

## Discussion

In this study, we found that not all independent variables had an effect on preventing hypertension problems in rural communities. The results revealed that internal factors influenced attitudes, subjective norms, and perceptions of behavioral control, while external factors only had a significant effect on subjective norms. Attitudes, through intentions, and perceptions of behavioral control, both through intentions and directly, significantly impacted hypertension prevention behavior. The novelty of this model lies in its identification of both the direct and indirect effects of these factors on hypertension prevention behavior in rural communities. This insight can guide the design of more targeted interventions, such as emphasizing the importance of strengthening personal experiences and tailoring media messages to improve understanding of hypertension prevention.

**Internal Factors (Experience)**: The study demonstrates that internal factors, particularly personal experiences, play a crucial role in shaping attitudes towards hypertension prevention in rural communities. For instance, 55.5% of hypertensive patients had positive experiences in managing their condition, which contributed to more proactive attitudes toward prevention. Past experiences with disease management significantly influence the formation of attitudes. The more experience an individual has, the more it is reflected in their attitude toward health behaviors. Experience in managing hypertension fosters awareness of the importance of prevention, ultimately leading to stronger attitudes and behaviors toward managing hypertension (15-18). Therefore, it is essential for healthcare providers, such as nurses, to better understand and consider these personal experiences in developing more effective prevention strategies.

Furthermore, internal factors also affect subjective norms—the beliefs individuals hold about the approval or disapproval of others regarding hypertension prevention. The experiences of individuals undergoing treatment help them recognize the influence of important figures, such as family and peers, in shaping their beliefs (19,20). The study suggests that motivation and health education efforts based on beliefs and past experiences can strengthen subjective norms and, in turn, improve hypertension prevention behaviors.

Moreover, experience also influences individuals' perceptions of their ability to control their health behaviors, particularly in managing hypertension. The study highlighted that individuals who had experienced health complications related to hypertension, such as stroke, were more likely to perceive themselves as capable of exerting control over their health behaviors. This increased perception of control promotes more active engagement in hypertension prevention (21,22).

**External Factors (Media Exposure)**: The study also explored the role of external factors, particularly media exposure, in influencing hypertension prevention behaviors. Surprisingly, media exposure did not have a significant impact on attitudes toward hypertension prevention. This could be attributed to the limited effectiveness of media in shaping beliefs and behaviors related to hypertension prevention in rural areas. The data indicated that a significant portion (30.8%) of hypertensive patients did not receive sufficient information through media outlets (23,24). The less interactive and indirect nature of media communication may have contributed to this low acceptance of health information.

However, media exposure did significantly influence subjective norms related to hypertension prevention. Hypertensive patients who had greater media exposure (69.2%) were more likely to adopt prevention-related behaviors. Health information disseminated through media channels can alter public perceptions and influence individuals to adopt preventive behaviors (25-27). Therefore, improving the quality and outreach of media campaigns on hypertension prevention could strengthen subjective norms and support healthier behaviors in rural communities.

Interestingly, media exposure did not affect perceptions of behavioral control in preventing hypertension. This suggests that media, while effective in shaping norms, may not be as successful in empowering individuals with the knowledge or confidence needed to control their health behaviors effectively. This finding is consistent with previous research suggesting that media interventions are more impactful in urban areas, where there is better access to health information and more trust in media sources (28-33).

**The role of intentions**: The study also revealed that attitudes and perceptions of control have a direct impact on behavioral intentions. This is particularly important for late adults (70.5% of the respondents), who tend to be more aware of the significance of health maintenance. Their life experiences and knowledge positively influence their intentions to prevent hypertension (34-35). Therefore, health education programs aimed at older adults in rural areas should emphasize the importance of prevention and increase their awareness of hypertension risks.

However, subjective norms, though important for shaping attitudes, did not significantly influence the intentions to prevent hypertension. This could be due to the low education levels (55.3%) among many participants, making it harder for them to grasp the concept of subjective norms and how they relate to their health behaviors (36-37).

Perceptions of behavioral control, on the other hand, significantly influenced hypertension prevention behavior. Patients with higher education levels (43.2%) and better knowledge about hypertension were more confident in their ability to take preventive actions (38). Providing accessible and targeted health education, particularly through classes and counseling, can enhance individuals' perceptions of control and increase their likelihood of engaging in preventive behaviors.

### Health Education and Community Empowerment

The findings highlight the need for tailored health education interventions, especially in rural communities, where access to healthcare services and information is limited. These interventions should focus on improving knowledge and awareness about hypertension, particularly for individuals with lower education levels (39,40). Empowering local community leaders, religious figures, and healthcare providers to disseminate information and encourage behavior change can significantly strengthen the collective effort to prevent hypertension in rural areas.

There were some limitations in this study. For example, challenges in accurately measuring blood pressure due to suboptimal facilities may have affected the results. Researchers addressed this by using alternative spaces that helped patients feel more at ease. Another limitation was the lack of interrater testing to ensure consistency between researchers and enumerators, which might have impacted the validity of the results regarding the sub-variables of internal and external factors. Addressing this limitation by conducting interrater tests prior to data collection would likely improve the validity of future studies.

In conclusion, this study identified key factors influencing hypertension prevention behavior in rural communities, emphasizing the importance of experience and media exposure. Internal factors, especially personal experiences with hypertension treatment, played a significant role in shaping attitudes, subjective norms, and perceptions of control. External factors, particularly media exposure, were more influential in shaping subjective norms. The findings suggest that targeted health education and community empowerment are critical in addressing hypertension prevention, particularly in rural settings.

Hypertensive patients are encouraged to increase their awareness and knowledge through local health education activities involving community leaders and healthcare providers. Future research could explore more specific cultural and educational factors to further enhance hypertension prevention efforts in rural communities.
